# An Intelligent Multi-Emissivity Infrared Temperature Correction Method for Substation Equipment Based on Semantic Segmentation

**DOI:** 10.3390/s26154754

**Published:** 2026-07-27

**Authors:** Sheng Han, Jialong Dong, Yafei Huang, Baifu Zhang

**Affiliations:** 1 Key Laboratory of Cleaner Intelligent Control on Coal & Electricity, Taiyuan University of Technology, Taiyuan 030600, Chinazhangbaifu@tyut.edu.cn (B.Z.); 2 State Key Laboratory of Disaster Prevention & Reduction for Power Grid, Changsha University of Science and Technology, Changsha 410114, China; huangyfei@csust.edu.cn

**Keywords:** temperature correction, substation equipment, infrared image, semantic segmentation

## Abstract

Emissivity is a critical parameter in infrared temperature measurement and varies significantly among different materials. Infrared thermography has been widely used for the inspection of substation equipment. However, substations contain a large number of devices with complex structures, making it impractical to assign a separate emissivity value to each device or component. This limitation can significantly affect temperature measurement accuracy. To address this issue, this paper proposes an intelligent multi-emissivity temperature correction method for infrared images of substation equipment. First, a temperature–emissivity correction function is established. Then, a total of 2189 infrared images of substation equipment are collected, and the main equipment components are annotated at the pixel level. Subsequently, an equipment component segmentation model based on DeepLabv3+ is trained. Finally, different emissivity values are assigned to different component regions for temperature correction, and corrected infrared pseudo-color images are regenerated. In the experiment, the temperature values before and after correction are compared with thermocouple measurements. In the present validation experiment, the average deviation between the corrected infrared temperature and the thermocouple measurement was reduced by 79.2% compared with that before correction.

## 1. Introduction

During long-term operation, substation equipment may experience abnormal heating caused by poor contact, insulation aging, partial discharge, and other defects [1,2]. A significant temperature rise is often an important indicator of such abnormal conditions. Owing to its non-contact and real-time characteristics, infrared thermography has become a widely used technique for condition monitoring and fault diagnosis of substation equipment [3,4,5,6]. However, the accuracy of infrared temperature measurement is strongly affected by the emissivity of the target surface [7,8]. Substation equipment usually consists of multiple components made of different materials, and these components may have significantly different emissivity values. In practical infrared inspection, the emissivity of a thermal imager is usually set to a fixed value, making it difficult to accurately represent the actual radiation characteristics of different components [9]. As a result, systematic temperature measurement errors may be introduced. Therefore, it is necessary to develop an intelligent correction method that can assign appropriate emissivity values to different components of substation equipment [10].

To improve the automation of infrared image analysis, image segmentation methods have been increasingly applied to infrared images of power equipment. Existing segmentation methods can generally be divided into traditional image processing-based methods and learning-based methods. Traditional methods usually rely on grayscale, temperature, or regional features [11,12,13]. For example, a temperature region division method based on superpixel segmentation and fuzzy clustering was proposed to segment infrared temperature regions [14]. Although such methods have relatively low computational complexity and certain real-time advantages, their performance is sensitive to parameter settings and can be easily affected by complex backgrounds and non-uniform temperature distributions. With the development of deep learning, learning-based methods have been gradually introduced into infrared image segmentation tasks [15,16,17,18]. An instance segmentation method based on visual feature reasoning was proposed and achieved improved segmentation performance [19]. However, most existing studies focus on overall equipment segmentation or high-temperature region extraction, while fine-grained segmentation of different components within the same equipment remains insufficient. This limits their applicability to temperature correction tasks that require component-specific emissivity assignment.

Infrared temperature measurement error analysis and correction have also attracted considerable research attention. From the perspective of radiative transfer theory, many studies have analyzed the sources of infrared temperature measurement errors [20]. The influencing factors have been classified into environmental factors, object-related factors, and thermal imager-related factors [21]. The influence of surface emissivity on infrared thermography accuracy has been theoretically analyzed [22], and experimental results have shown that the emissivity setting of a thermal imager should match the surface material of the measured object [23]. In terms of correction methods, an infrared temperature calibration method based on Bayesian inference was proposed, in which an improved infrared radiation model and joint maximum a posteriori estimation were used to reconstruct model parameters and temperature distribution [24]. In another study, targets were first extracted using a segmentation network and mask processing; the images were then enhanced using a convolutional neural network, and the temperature distribution was finally obtained through radiometric calibration [25]. These studies have improved the accuracy of infrared temperature measurement from different perspectives. Nevertheless, most existing correction methods still do not sufficiently consider the spatial variation in emissivity among different components within substation equipment.

In summary, infrared thermography has been widely used for condition monitoring of power equipment, and existing studies have made progress in infrared image segmentation and temperature correction. However, for substation equipment with complex structures and multiple surface materials, the fixed-emissivity setting commonly used in infrared temperature measurement cannot accurately describe the radiation characteristics of different components. Existing methods still lack the ability to automatically identify component-level emissivity differences and perform region-specific temperature correction.

To overcome these limitations, this paper proposes an intelligent multi-emissivity temperature correction method for substation equipment based on semantic segmentation. The main contributions of this work are as follows. First, an infrared image dataset of substation equipment is constructed, and the main equipment components are annotated at the pixel level. Second, a DeepLabv3+ semantic segmentation model is trained to identify different equipment components in infrared images. Third, different emissivity values are assigned to different component regions based on the segmentation results, and the original infrared temperature matrix is corrected pixel by pixel. Finally, the corrected temperature results are regenerated as infrared pseudo-color images and validated through comparative experiments with thermocouple measurements.

## 2. Multi-Emissivity Temperature Correction Method

The basic components of an infrared thermal imager include an infrared lens, a focal plane detector, a signal processing module, and an image output system [26,27]. The response characteristics of infrared detectors vary with detector type and spectral band. According to their operating wavelength ranges and application scenarios, thermal imagers can generally be classified into short-wave (0.9–3 μm), mid-wave (3–5 μm), and long-wave (8–14 μm) systems [28,29,30]. Among these, long-wave infrared thermal imagers are widely used for temperature measurement of substation equipment because of their relatively high atmospheric transmittance and low background interference [31,32].

The actual surface temperature of an object is typically calculated using Equation (1):

(1) $$T={\left\{\frac{1}{\varepsilon }\left[\frac{1}{\tau_{a}}T_{r}^{n}(1-\varepsilon )T_{u}^{n}-\left(\frac{1}{\tau_{a}}-1\right)T_{a}^{n}\right]\right\}}^{\frac{1}{n}}$$

where *T* represents the actual surface temperature of the object, *ε* is the object emissivity, *τ*_a_ is the atmospheric spectral transmittance, *T*_r_ is the object surface radiation temperature, *T*_u_ is the ambient temperature, *T*_a_ is the atmospheric temperature, and n depends on the spectral band of the thermal imager. Specifically, n = 9.255 for the 2–5 μm band and n = 3.988 for the 8–14 μm band.

Emissivity *ε* is a dimensionless parameter that describes the ability of a material surface to emit thermal radiation relative to an ideal blackbody at the same temperature.

In practical substation inspections, the measurement distance is typically about 3–8 m. At this relatively short distance, and under stable atmospheric conditions, *τ*_a_ can be approximated as 1, and Equation (1) can be simplified as Equation (2):

(2) $$T={\left\{\frac{1}{\varepsilon }\left[T_{r}^{n}-(1-\varepsilon )T_{u}^{n}\right]\right\}}^{\frac{1}{n}}$$

In substations, electrical equipment is usually installed with sufficient spacing to satisfy air insulation requirements. Under stable ambient conditions, the influence of the ambient temperature term becomes relatively small, and *T*_u_/*T* can be neglected as an approximation. *T* can be further expressed as Equation (3):

(3) $$T=\frac{T_{r}}{\sqrt[{n}]{\varepsilon }}$$

It should be noted that the above simplification is applicable because substation infrared inspections are usually conducted under relatively stable weather conditions. Severe environmental conditions, such as heavy rain, strong wind, dense fog, and intense direct solar radiation, are generally avoided to reduce environmental interference. Under such inspection conditions, the atmospheric transmittance is relatively high, and the amplification effect introduced by 1/*τ*_a_ is limited. Therefore, this simplification is not applicable to all infrared temperature measurement scenarios.

In practical infrared inspection, a single emissivity value is usually set in the thermal imager. However, substation equipment is often composed of multiple components with different surface materials, and these components may have different emissivity values. Therefore, using a fixed emissivity value for the entire infrared image may introduce temperature measurement errors in different component regions.

According to the Stefan–Boltzmann law, the radiant energy emitted by an object is proportional to $\varepsilon \cdot T^{4}$ [33]. Here, n = 3.988 was approximated as n = 4 in Equation (3). The difference introduced by this approximation is negligible under the emissivity range considered in this study. Under the same received radiation signal, the corrected temperature is therefore related to the emissivity setting. Based on this relationship, emissivity-based temperature correction can be expressed as Equation (4):

(4) $$T_{c}={\left(\frac{\varepsilon_{0}}{\varepsilon_{c}}\right)}^{\frac{1}{4}}\cdot T_{0}$$

where *T*_c_ represents the infrared temperature measurement result after emissivity–temperature correction, *ε*_0_ is the emissivity originally set in the thermal imager, *ε*_c_ is the emissivity corresponding to the surface material of the corrected equipment component, and *T*_0_ is the temperature measurement result before correction. The emissivity–temperature correction parameter *C* is defined as:

(5) $$C={\left(\frac{\varepsilon_{0}}{\varepsilon_{c}}\right)}^{\frac{1}{4}}$$

To further illustrate the influence of emissivity variation on the temperature correction result, the relative change in the correction coefficient under different emissivity settings was calculated, as shown in Table 1. The rows represent the initial emissivity setting ***ε*_0_**, and the columns represent the assigned emissivity ***ε*_*c*_** after component identification. The values in the table represent the relative change in the correction coefficient, calculated as (*C* − 1) × 100%. A positive value indicates that the corrected temperature increases after emissivity adjustment, while a negative value indicates that the corrected temperature decreases.

As shown in Table 1, when the difference between the preset emissivity and the assigned emissivity is small, the correction coefficient changes only slightly. However, when the emissivity difference is large, the influence on the correction coefficient becomes significant. This indicates that accurate component identification and emissivity assignment are important for multi-emissivity temperature correction.

**Figure 1:**
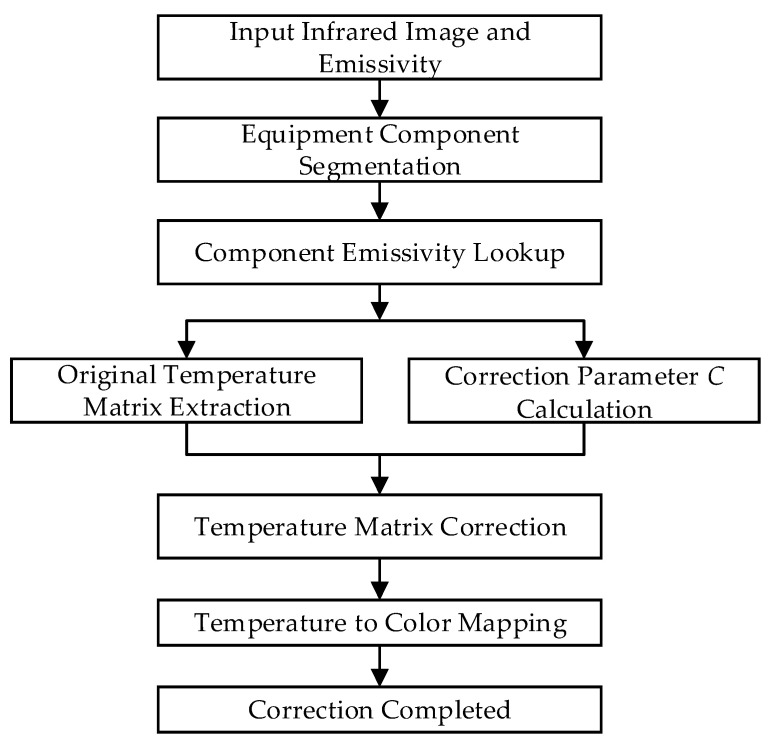
Flowchart of the proposed multi-emissivity temperature correction method.

Based on the above analysis, this paper proposes a multi-emissivity temperature correction scheme, as illustrated in Figure 1. In this scheme, the infrared image is first segmented into different equipment component regions. Then, the corresponding emissivity value is assigned to each component according to its surface material. Finally, the original infrared temperature matrix is corrected at the pixel level, and the corrected infrared pseudo-color image is generated. In this way, the proposed method enables region-specific emissivity assignment and temperature correction for substation equipment with multiple surface materials.

## 3. Component Segmentation Model for Substation Equipment

### 3.1. Introduction to the DeepLabv3+ Model

Image segmentation models can generally be divided into semantic segmentation and instance segmentation. The main difference between them lies in whether different instances belonging to the same category are distinguished. Since this study focuses on emissivity–temperature correction for substation equipment, the objective is to identify component regions with different emissivity values rather than to distinguish individual instances with the same emissivity. Therefore, semantic segmentation is adopted in this study.

DeepLabv3+ is a semantic segmentation model proposed by Google [34]. It is designed to achieve fine segmentation of target regions while maintaining relatively high computational efficiency, making it suitable for objects with complex boundary structures. Infrared images of substation equipment usually contain complex component layouts and repeated structural patterns. Therefore, the multi-scale feature extraction capability of DeepLabv3+ is well suited for component-level segmentation of substation equipment.

**Figure 2:**
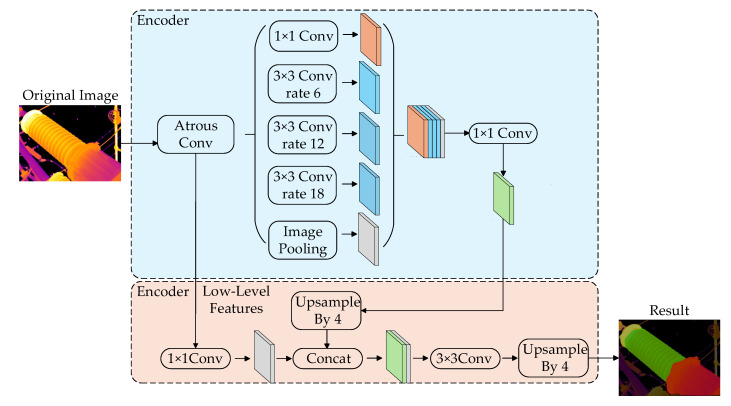
The basic structure of the DeepLabv3+ model.

The overall architecture of the model is shown in Figure 2. In this study, Xception was adopted as the backbone network of DeepLabv3+ for feature extraction. The model mainly consists of two parts: an encoder and a decoder. The encoder is responsible for extracting multi-scale features. By introducing atrous convolution and the Atrous Spatial Pyramid Pooling (ASPP) module, the model can capture spatial information at different scales. The decoder is responsible for boundary refinement. It integrates low-level detailed features with high-level semantic features, thereby improving the accuracy of segmentation boundaries.

### 3.2. Infrared Image Dataset

**Figure 3:**
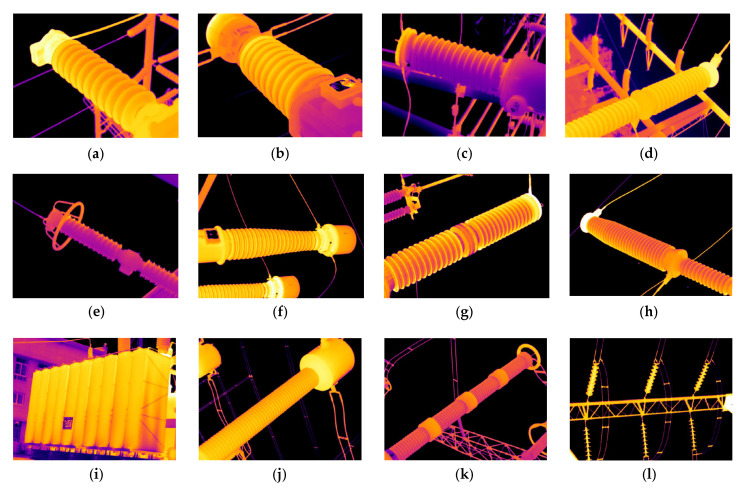
Original data and equipment categories: (**a**) 110 kV arrester; (**b**) 110 kV CT; (**c**) 110 kV PT (**d**) 110 kV breaker; (**e**) 220 kV arrester; (**f**) 220 kV CT; (**g**) 220 kV PT; (**h**) 220 kV breaker; (**i**) transformer; (**j**) 500 kV CT; (**k**) 500 kV PT; (**l**) insulator string.

In this study, a total of 2189 infrared images of substation equipment in actual operation were collected and organized. The dataset covers seven common types of substation equipment, including potential transformers (PT), current transformers (CT), circuit breakers, and power transformers. The images were acquired using a FLIR T630 handheld infrared thermal camera (FLIR Systems, Inc., Wilsonville, OR, USA), with the emissivity uniformly set to 0.95. Figure 3 shows the collected samples and the equipment classification scheme used in this study. Table 2 summarizes the number of samples in each category and the annotated components.

For the components listed in Table 2, pixel-level annotation was performed using the LabelMe 5.5, and the annotation results were organized in the Visual Object Classes (VOC) format for model training. In this study, equipment components with the same material and similar shapes were assigned to the same label. In particular, insulator strings in substations are usually located far from the imaging position, making it difficult to capture their small steel parts. Therefore, only the ceramic parts of insulator strings were annotated.

## 4. Experimental Results and Analysis

The proposed method consists of three main sequential steps: component segmentation, emissivity lookup, and temperature correction. These steps have different effects on the final corrected imaging results. The component segmentation result determines the spatial regions and categories of different equipment components, thereby affecting the correctness of emissivity assignment and the spatial consistency of the regenerated infrared pseudo-color image. The emissivity lookup mechanism maps the segmented component labels to the corresponding emissivity values according to material properties. The temperature correction formula is then applied pixel by pixel to the original infrared temperature matrix. Therefore, the experiments in this section are designed to evaluate the segmentation performance, verify the component-based emissivity assignment, and assess the final temperature correction accuracy using thermocouple measurements.

### 4.1. Training and Testing of the Segmentation Model

In this study, the substation equipment component segmentation model was trained based on DeepLabv3+ and the constructed dataset. The experimental platform was equipped with two NVIDIA GeForce RTX 4090 GPUs. PyTorch 2.13.0 was used as the deep learning framework, and Python 3 was used as the programming language.

During training, stochastic gradient descent (SGD) was used as the optimizer. The initial learning rate was set to 7 × 10^−3^, the batch size was set to 16, and the number of training epochs was set to 200. The dataset was divided into a training set and a validation set at a ratio of 7:3. Model performance was evaluated on the validation set every 5 epochs, and the training loss, validation loss, and mean intersection over union (mIoU) were recorded.

**Figure 4:**
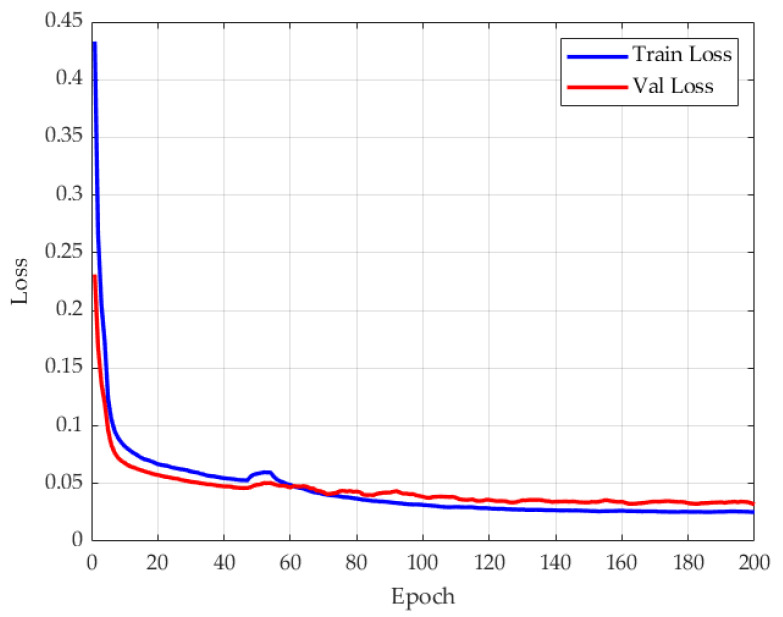
Training and validation loss curves.

The cross-entropy loss was adopted to measure the pixel-level classification error between the predicted segmentation results and the manually annotated labels. Figure 4 shows the corresponding loss curves. As shown in Figure 4, the loss values gradually became stable when the model was trained for approximately 140 epochs. After training was completed, the training loss converged to 0.025, while the validation loss was 0.031, indicating that the model achieved good convergence without obvious overfitting.

**Figure 5:**
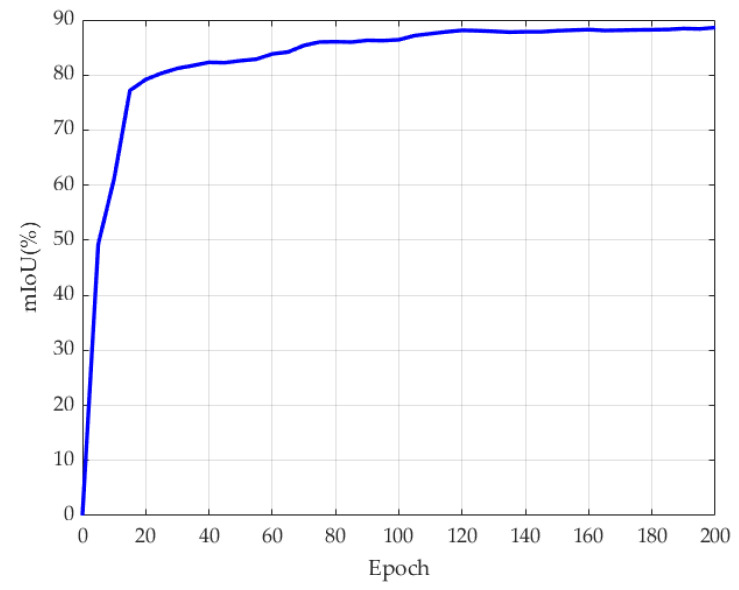
Curve of mIoU during training.

In addition to the loss curves, the mIoU focuses on the spatial overlap between the predicted regions and the ground truth regions, and thus directly reflects the quality of the segmentation results at the region level. As shown in Figure 5, the mIoU gradually increased as training proceeded and tended to stabilize after approximately 140 epochs. After training was completed, the mIoU reached 88.62%, indicating that the model could effectively capture the image features of different components of substation equipment.

To provide a more detailed evaluation of the component segmentation performance, additional pixel-level metrics were calculated on the validation set. Since the validation set contains manually annotated ground-truth masks, precision, recall, F1-score, and IoU were calculated for each annotated equipment component. Table 3 summarizes the segmentation results for different equipment types, component categories, and surface materials. These results provide a quantitative basis for evaluating whether the segmentation model can correctly identify component regions with different emissivity values.

As shown in Table 3, the average precision, recall, F1-score, and IoU reach 94.91%, 94.44%, 95.00%, and 85.63%, respectively, indicating that the model can effectively identify most equipment components. It should be noted that this difference is not caused only by the material itself, but is closely related to the structural characteristics of the corresponding components. Components made of electro-porcelain generally achieve high segmentation performance, which may be attributed to their distinctive corrugated contours and structural features in infrared images. In contrast, relatively lower IoU values are observed for some metal components, such as the fuel tank, transformer body, and metal base of surge arresters. This is because they usually have more variable shapes and often contain attached structures or surface accessories, such as connectors, bolts, and nameplates. These small structures increase the complexity of the component appearance and make it more difficult for the model to distinguish the main component region from adjacent or attached structures. Therefore, local boundary deviations and misclassifications are more likely to occur for these metal components. The average IoU values of stainless-steel and iron components are 84.32% and 82.11%, respectively, which are lower than that of electro-porcelain components. Overall, these results provide a more detailed evaluation of the segmentation model and support its use for subsequent component-level emissivity assignment.

**Figure 6:**
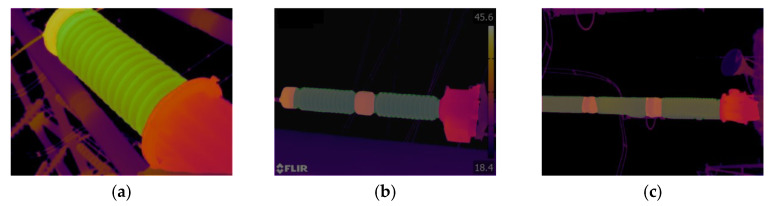
Component segmentation test results for different types of equipment: (**a**–**c**) samples of different types of equipment, the segmentation results were overlaid on the original infrared images for comparison.

After training, the equipment component segmentation model was evaluated on the test set. The test set contained 100 infrared images for each of the five types of equipment, and these images were not included in the training data. As shown in Figure 6, the segmentation results were overlaid on the original infrared images for comparison. It can be observed that the proposed model can generally segment the equipment components effectively, while the main difficulty lies in the boundary and connection regions between different components. As shown in Figure 6a, when the imaging distance is relatively short, the extracted contours are smooth and accurate. However, when the imaging distance increases, as shown in Figure 6c, deviations may occur at the segmentation boundaries.

**Figure 7:**
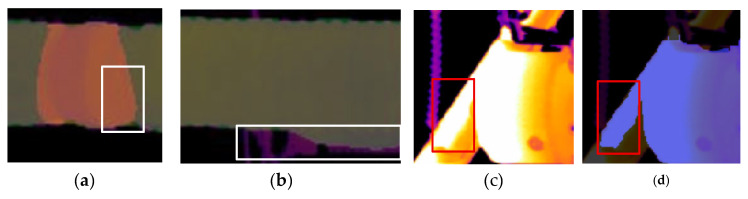
Typical error-prone cases in component segmentation: (**a**) boundary deviation between adjacent components under long-distance imaging; (**b**) boundary overflow caused by background equipment with similar structural features; (**c**,**d**) segmentation error caused by thermal saturation.

Figure 7 shows several typical error-prone cases in component segmentation. The first type is boundary deviation under long-distance imaging, as shown in Figure 7a. In this case, the boundary between adjacent components may shift locally, resulting in incorrect emissivity assignment for pixels near the material interface. According to the theoretical analysis in Table 1, the influence of such errors depends on the emissivity difference between the incorrectly assigned material and the actual material. When adjacent components have similar emissivity values, the influence on the correction coefficient is relatively limited. However, when the emissivity difference is large, the local correction deviation may become significant. For example, if an electro-porcelain component is incorrectly identified as stainless steel, the correction coefficient may be overestimated by 54.85%. Conversely, if a stainless-steel component is incorrectly identified as electro-porcelain, the correction coefficient may be underestimated by 35.42%. Therefore, boundary errors between metal components and insulating components, such as a stainless-steel flange and an electro-porcelain bushing, may introduce obvious local temperature discontinuities in the regenerated infrared pseudo-color image.

The second type is boundary overflow caused by background equipment with similar structural features, as shown in Figure 7b. This error occurs because the model may extract structures in the background that are visually similar to the target equipment components. However, since background equipment is not the target region for infrared temperature analysis, this type of error has limited influence on the final temperature correction result. The third type is caused by thermal saturation, as shown in Figure 7c,d. When the upper temperature limit of the infrared image is lower than the actual equipment temperature, saturated white regions may become connected in the image. As a result, the boundaries between different components become difficult to distinguish, leading to incorrect segmentation. This indicates that proper infrared imaging parameter settings need to be adjusted before temperature correction.

Overall, these error cases show that boundary errors remain an important limitation of the current framework. Therefore, boundary refinement, post-processing, and uncertainty-aware segmentation should be further investigated in future work. For example, more refined boundary annotations and boundary-aware learning strategies may improve the model’s ability to distinguish adjacent components. Post-processing methods, such as morphological operations, connected-region filtering, or conditional random fields, may help reduce small isolated misclassified regions. In addition, uncertainty-aware segmentation can be used to identify low-confidence pixels near component boundaries, where soft emissivity assignment or confidence-weighted temperature correction may be more appropriate than hard label assignment.

Another promising direction is visible–infrared multimodal fusion. Because infrared thermography is limited by its imaging mechanism and sensor resolution, fine details at material interfaces are sometimes not clearly represented in infrared images. Under good illumination conditions, visible-light images can provide higher spatial resolution and richer color and texture information at relatively low cost, which may help improve boundary discrimination. However, visible-light images cannot directly replace infrared images in all inspection scenarios, because infrared inspections are often performed at dusk or at night to reduce the influence of solar radiation. Therefore, fusing visible and infrared information may be a more practical approach, in which visible images assist boundary perception when available, while infrared images provide the temperature information required for correction.

### 4.2. Comparison of Semantic Segmentation Models

To further evaluate the rationality of selecting DeepLabv3+ as the component segmentation model, comparative experiments were conducted with three representative semantic segmentation models, namely U-Net, HRNet, and SegFormer-B0. These models represent different segmentation architectures. U-Net is a classical encoder–decoder CNN with skip connections, HRNet maintains high-resolution feature representations throughout the network, and SegFormer-B0 is a lightweight Transformer-based semantic segmentation model.

For a fair comparison, all models were trained and evaluated using the same dataset partition, annotation categories, input image resolution, number of training epochs, batch size, and evaluation metrics. The original infrared images were uniformly resized to 640 × 640 before being input into the network. The number of training epochs was set to 200, and the batch size was set to 16 for all compared models. Since these models have different architectural designs and commonly used training strategies, their representative default configurations were retained. Specifically, SegFormer-B0 was trained using AdamW, while U-Net, HRNet, and DeepLabv3+ were trained using SGD. The learning-rate parameters followed the default settings of the corresponding implementations and were not forcibly unified. Therefore, the comparison was conducted under a unified data and evaluation protocol while preserving the typical training configuration of each model.

As shown in Table 4, DeepLabv3+ achieves the best overall segmentation performance among the compared models, with an mIoU of 85.63%, precision of 94.91%, recall of 94.44%, and F1-score of 95.00%. HRNet obtains a very close mIoU of 85.52%, indicating that maintaining high-resolution feature representations is also effective for component segmentation of substation equipment. However, HRNet has the largest number of parameters, reaching 68 M, while its precision, recall, and F1-score are lower than those of DeepLabv3+. U-Net achieves an mIoU of 81.36%, but its ability to model multi-scale contextual information is relatively limited, which may affect the segmentation of repeated and scale-varying equipment components. SegFormer-B0 has the smallest number of parameters, only 3.7 M, showing clear advantages in model lightweightness. However, its segmentation performance is the lowest in this experiment, with an mIoU of 75.07%. This may be because the infrared images in this study contain relatively weak texture information, and the differences among components are mainly reflected in shape, contour, and local structural boundaries. Under the current dataset and training conditions, the lightweight Transformer structure of SegFormer-B0 does not show an advantage over CNN-based models. Overall, DeepLabv3+ provides the best balance between segmentation accuracy and model complexity in this task, and is therefore adopted as the component segmentation model in the proposed multi-emissivity temperature correction framework.

It should be noted that the proposed framework does not depend on a specific segmentation architecture. The semantic segmentation model is used to generate component regions and the corresponding emissivity map. After the emissivity map is obtained, the subsequent emissivity lookup and temperature correction process is deterministic. Therefore, different segmentation models mainly affect the correction result through the accuracy of component recognition and emissivity assignment. The benchmark comparison in this section provides a basis for model selection and also indicates that other segmentation models can be integrated into the framework according to specific engineering requirements.

### 4.3. Multi-Emissivity Correction Imaging Test

**Figure 8:**
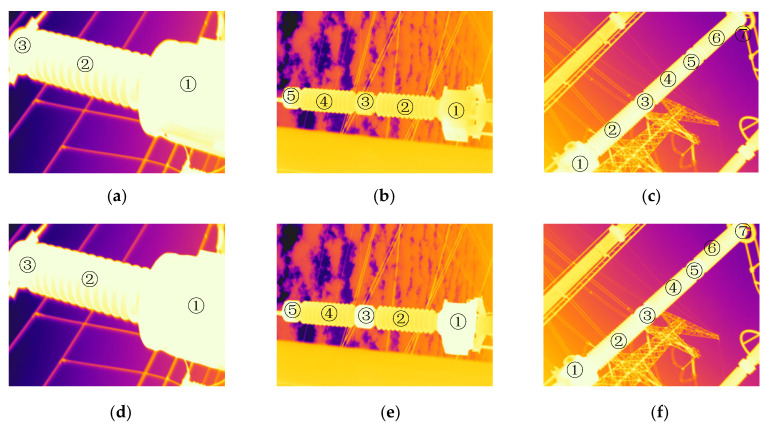
Multi-emissivity correction imaging test: (**a**–**c**) original images; (**d**–**f**) corrected images, and numbers represent different structural recognition results.

Following the workflow shown in Figure 1, the test set images were corrected and regenerated as infrared pseudo-color images. The infrared temperature matrix in CSV format and the original global emissivity setting were extracted using FLIR Tools (version 6.4). The results are shown in Figure 8.

In the original images, the emissivity was set to 0.95 during image acquisition. According to Table 2, the surfaces of the flange and electromagnetic unit in the PTs are both made of stainless steel, and their emissivity should be set to 0.16, corresponding to labels 3 and 1, respectively. The surface of the bushing is made of ceramic, with an emissivity of 0.92. The correction parameters were calculated using Equation (5). The correction parameter *C* for the flange and electromagnetic unit was 1.561, while that for the bushing was 1.008.

Table 5 lists the temperature data of each component in Figure 7. For each comparison, the temperature values were extracted from the same coordinate point in the images before and after correction. For the samples listed in Table 5, the average temperature difference between the original and corrected values for all components was 9.20 °C. The magnitude of this difference is related to the emissivity setting of the infrared camera. As mentioned above, the emissivity was uniformly set to 0.95 in this experiment. The closer the material emissivity is to the preset value, the smaller the temperature difference introduced by correction.

For the flange and electromagnetic unit, the actual emissivity of the surface material differs considerably from the preset value, resulting in a large temperature difference before and after correction. The average temperature difference between the original and corrected values was 15.19 °C for the flange and 15.18 °C for the electromagnetic unit. In contrast, the emissivity correction for the bushing was relatively small; therefore, the temperature difference before and after correction was also small, with an average value of only 0.21 °C. Correction tests were also performed on all samples in the dataset, and the average temperature difference between the original and corrected values was 8.53 °C.

### 4.4. Correction Accuracy Test

**Figure 9:**
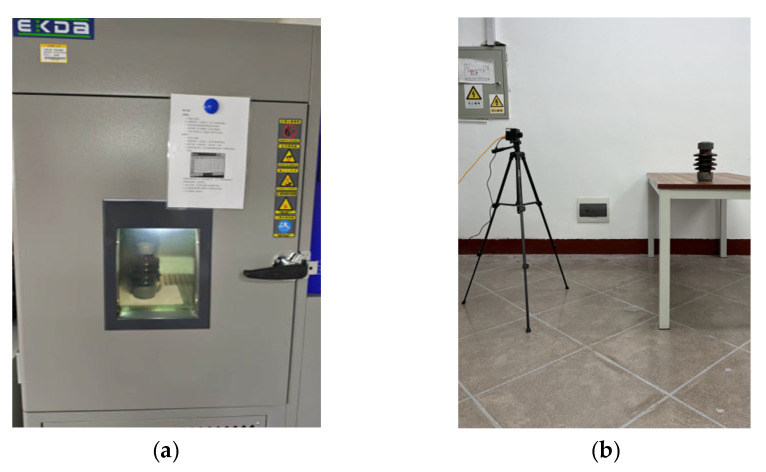
Test site for correction accuracy verification: (**a**) heating the sample in a constant temperature chamber; (**b**) temperature measurement and correction.

To verify the effectiveness and accuracy of the proposed method, a real-time imaging test was carried out using an infrared temperature measurement module. As shown in Figure 9, the insulator sample was first heated in a thermostatic chamber at a set temperature of 60 °C for 30 min. The sample was then removed from the chamber, and its temperature was measured using a Yoseen M infrared module (Yoseen Infrared Co., Ltd., Wuhan, China). The temperature data were corrected in real time on the computer. This is a secondary development implementation based on the SDK provided by the supplier. Finally, the corrected temperature values were compared with the measurements obtained using a thermocouple thermometer.

**Figure 10:**
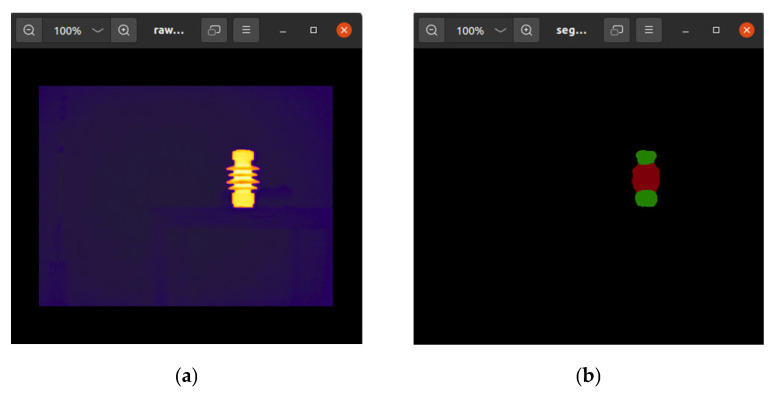
Original infrared imaging result and real-time segmentation result: (**a**) Original infrared image of post insulator; (**b**) Infrared image segmentation results of post insulators.

As shown in Figure 10a, the imaging quality of the infrared module used in this test differs significantly from that of the FLIR T630 used to construct the dataset. This module differs from the FLIR cameras in imaging quality, edge sharpness, and image details, and the experiment was performed on an indoor background. Nevertheless, as shown in Figure 10b, the proposed model can still accurately segment the equipment components.

Since the segmentation module is based on DeepLabv3+ without major structural modification, the computational cost of the proposed framework is mainly determined by the standard segmentation network. The additional multi-emissivity correction process introduces limited overhead. Specifically, after segmentation, the emissivity lookup is implemented through label-based mapping, and the temperature correction is performed as a pixel-wise operation on the infrared temperature matrix. For a 640 × 480 temperature matrix, this process only requires generating the corresponding correction coefficient map and applying pixel-wise calculation. The pseudo-color reconstruction also introduces limited computational cost.

The test platform was equipped with an Intel i7-13700K CPU at 3.40 GHz and a single NVIDIA GeForce RTX 4090 GPU. During the test, the temperature stream and the H.264 image stream were simultaneously acquired through the SDK. The image stream was decoded and input into the segmentation model to generate the temperature correction mask. According to the timestamp, the correction mask was matched with the corresponding temperature frame in the temperature stream. Then, temperature correction was performed on the matched temperature matrix, and the corrected temperature data were reconstructed into an infrared pseudo-color image using the predefined pseudo-color scheme. The complete real-time correction system achieved a stable processing speed of 25 FPS, which is consistent with the imaging frame rate of the infrared module. This processing speed includes stream acquisition, image segmentation, mask matching, temperature correction, and pseudo-color image reconstruction, rather than only the inference speed of the segmentation model.

**Figure 11:**
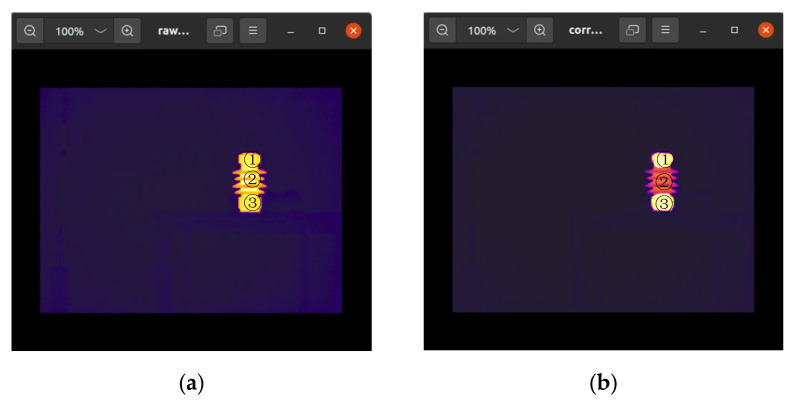
Imaging results of correction accuracy test: (**a**) Original infrared image of post insulator; (**b**) Infrared image of post insulator after correction, and numbers represent different structural recognition results.

The emissivity of the infrared module used in this test was set to 0.97. According to Table 2, the surface material of the flange is iron, with an emissivity of 0.44, while the surface material of the insulator body is ceramic, with an emissivity of 0.92. According to Equation (5), the emissivity–temperature correction parameters *C* for the flange and bushing were calculated as 1.22 and 1.01, respectively. Figure 11b shows the reconstructed infrared pseudo-color image generated from the corrected temperature values. Since the FLIR pseudo-color scheme was adopted for image reconstruction, its color style is slightly different from the original image.

**Figure 12:**
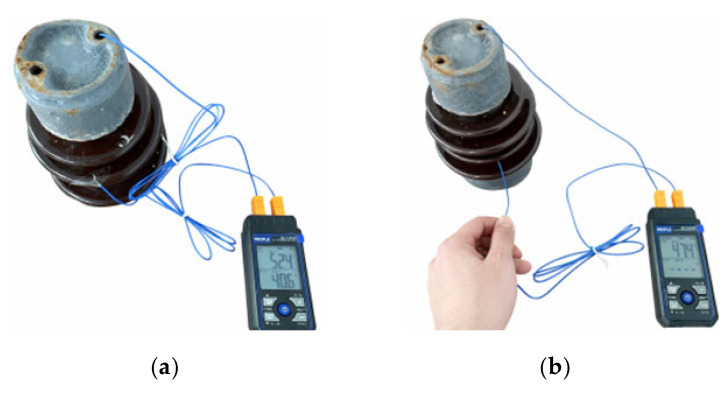
Temperature measurement results using thermocouple thermometer: (**a**) Temperature measurement results of flange ① and insulator body ②; (**b**) Temperature measurement result of flange ③.

To evaluate the correction performance in this test, a high-precision thermocouple thermometer was used to measure the temperature of the post insulator as a reference during infrared temperature acquisition. The field test setup is shown in Figure 12.

The test results are shown in Table 6. For the flange components, the default emissivity setting differs considerably from the actual emissivity value. As a result, the temperature differences before and after correction were 11.65 °C, 2.24 °C and 10.82 °C, respectively. This observation is consistent with the experimental results shown in Table 5.

For the three measured components, the average deviation between the corrected infrared temperature and the actual temperature was 1.31 °C. Compared with the value before correction, the average deviation decreased by 6.29 °C, corresponding to an error reduction of 79.2%. The corrected infrared temperatures of the two flange components were both higher than the actual values, with an average error of 1.46 °C. In contrast, the corrected infrared temperature of the bushing insulator component was 1.01 °C lower than the actual value. Although it represents the worst result among the three measured components, the error is still reduced by 68.9% compared with that before correction.

## 5. Conclusions

Infrared thermography has been widely used in condition monitoring and fault diagnosis of substation equipment. However, infrared temperature measurement is easily affected by the emissivity of the equipment surface, which may lead to measurement errors. In practical applications, most infrared thermal cameras usually use a single emissivity setting and cannot assign different emissivity values to different equipment components for temperature correction. To address this problem, this paper proposes an intelligent multi-emissivity temperature correction method based on semantic segmentation.

In this study, the constructed dataset was used to train a DeepLabv3+ semantic segmentation model, enabling fine segmentation of different components of substation equipment. Based on the segmentation results, the actual emissivity values were automatically obtained through table lookup, and the corresponding correction parameters were calculated. The original temperature matrix of the infrared image was then corrected pixel by pixel to obtain the final temperature correction results and generate the corrected infrared pseudo-color image.

The test results show that the average accuracy of equipment component segmentation reaches 95%. Comparative tests were conducted using contact thermocouple measurements as the reference. In this validation experiment, the average deviation between the corrected infrared temperature and the thermocouple measurement was reduced by 79.2% compared with that before correction. Therefore, the current experiment verifies the feasibility and accuracy trend of the proposed correction method, while more validation tests involving different equipment, materials, and temperature conditions are still needed in future work. The proposed method realizes intelligent temperature correction considering multiple emissivity values, which is important because emissivity is the dominant factor affecting infrared temperature measurement accuracy in most cases. In future work, intelligent correction methods for other parameters, such as measurement distance, can be further investigated to further improve temperature measurement accuracy. In addition, boundary refinement, uncertainty-aware emissivity assignment, and visible–infrared multimodal fusion will be explored to reduce local correction errors near component boundaries and improve the robustness of the proposed framework.

## Figures and Tables

**Table 1:** The relative deviation of the correction coefficient (%).

*ε*_0_/*ε*_*c*_	0.1	0.2	0.3	0.4	0.5	0.6	0.7	0.8	0.9	1.0
0.1	0.0	−15.9	−24.0	−29.3	−33.1	−36.1	−38.5	−40.5	−42.3	−43.8
0.2	18.9	0.0	−9.6	−15.9	−20.5	−24.0	−26.9	−29.3	−31.3	−33.1
0.3	31.6	10.7	0.0	−6.9	−12.0	−15.9	−19.1	−21.7	−24.0	−26.0
0.4	41.4	18.9	7.5	0.0	−5.4	−9.6	−13.1	−15.9	−18.4	−20.5
0.5	49.5	25.7	13.6	5.7	0.0	−4.5	−8.1	−11.1	−13.7	−15.9
0.6	56.5	31.6	18.9	10.7	4.7	0.0	−3.8	−6.9	−9.6	−12.0
0.7	62.7	36.8	23.6	15.0	8.8	3.9	0.0	−3.3	−6.1	−8.5
0.8	68.2	41.4	27.8	18.9	12.5	7.5	3.4	0.0	−2.9	−5.4
0.9	73.2	45.6	31.6	22.5	15.8	10.7	6.5	3.0	0.0	−2.6
1.0	77.8	49.5	35.1	25.7	18.9	13.6	9.3	5.7	2.7	0.0

**Table 2:** Number of samples and annotated components.

Equipment Type	Voltage (kV)	Sample Size	Component	Surface Material	Emissivity	Label
PT	110/220/500	122/147/139	Flange	Stainless steel	0.16	3
			Bushing	Electroporcelain	0.92	2
			electromagnetic unit	Stainless steel	0.16	1
CT	110/220/500	136/109/125	Bushing	Electroporcelain	0.902	2
			Fuel tank	Iron	0.44	4
Arrester	110/220	117/126	Flange	Stainless steel	0.16	5
			Housing	Electroporcelain	0.92	2
			Base	Stainless steel	0.16	6
Transformer	-	369	Body	Stainless steel	0.16	7
			Bushing	Electroporcelain	0.92	2
Insulator String	-	365	Insulator	Electroporcelain	0.92	11
Post Insulator	9	165	Flange	Iron	0.44	8
			Insulator body	Electroporcelain	0.92	9
Breaker	110/120	117/152	Flange	Stainless steel	0.16	10
			Bushing	Electroporcelain	0.92	2

**Table 3:** Component-level segmentation performance of substation equipment (%).

Type	Component	Material	Precision	Recall	F1-Score	IoU
PT	Flange	Stainless steel	94.36	93.14	93.74	84.26
	Bushing	Electroporcelain	95.88	95.02	95.94	77.60
	Electromagnetic unit	Stainless steel	93.42	92.51	92.96	83.70
CT	Bushing	Electroporcelain	98.40	96.96	97.68	95.81
	Fuel tank	Iron	90.81	90.08	90.93	75.20
Arrester	Flange	Stainless steel	97.87	97.97	98.42	81.97
	Housing	Electroporcelain	96.54	96.97	97.26	91.77
	Base	Stainless steel	90.27	89.68	89.97	85.60
Transformer	Body	Stainless steel	94.55	94.84	95.20	78.66
	Bushing	Electroporcelain	96.64	96.17	96.90	86.16
Post Insulator	Flange	Iron	94.31	93.22	93.76	89.02
	Insulator body	Electroporcelain	95.75	95.03	95.88	89.59
Insulator String	Insulator	Electroporcelain	94.66	94.30	94.97	81.77
Breaker	Flange	Stainless steel	91.53	91.83	92.18	91.71
	Bushing	Electroporcelain	98.67	98.87	99.27	91.62
Average	-	-	94.91	94.44	95.00	85.63

**Table 4:** Comparison of mIoU results for different semantic segmentation models.

Model	Params (M)	mIoU (%)	Precision (%)	Recall (%)	F1-Score (%)
U-Net	29	81.36	88.24	87.72	87.98
HRNet-W48	68	85.52	92.62	91.92	92.27
SegFormer-B0	3.7	75.07	80.17	79.19	79.68
DeepLabv3+	42	85.63	94.91	94.44	94.67

**Table 5:** Temperature values of the multi-emissivity correction imaging test (°C).

Sample	Component	Identification	Original	Corrected	Difference
1	Flange③	Correct	35.9	56.04	20.14
	Bushing②	Correct	32.8	33.06	0.26
	Electromagnetic unit①	Correct	33.9	52.92	19.02
2	Flange⑤	Correct	19.2	29.97	10.77
	Flange③	Correct	18.4	28.72	10.32
	Bushing④	Correct	17.0	17.13	0.13
	Bushing②	Correct	17.1	17.23	0.13
	Electromagnetic unit①	Correct	16.1	25.13	9.03
3	Flange⑦	Correct	30.3	47.29	16.99
	Flange⑤	Correct	28.6	44.64	16.04
	Flange③	Correct	30.1	46.98	16.88
	Bushing⑥	Correct	29.9	30.14	0.24
	Bushing④	Correct	30.4	30.64	0.24
	Bushing②	Correct	30.1	30.34	0.24
	Electromagnetic unit①	Correct	31.2	48.70	17.50

**Table 6:** Comparison of infrared and thermocouple temperature measurements (°C).

Type	Component	Original	Corrected	Thermocouple	Original Error	Corrected Error
Post insulator	Flange①	41.60	53.25	52.4	−10.80	0.85
	Insulator②	37.35	39.59	40.6	−3.25	−1.01
	Flange③	38.65	49.47	47.4	−8.75	2.07

## Data Availability

The full dataset presented in this study is available from the corresponding author upon reasonable request. Due to operational and confidentiality restrictions related to substation equipment, the full infrared image dataset is not publicly available. A representative sample dataset, including original infrared images and semantic segmentation annotations, has been provided as Supplementary Material to illustrate the image format, annotation format, directory structure, and class definitions.

## Supplementary Materials

The following supporting information can be downloaded at: https://www.mdpi.com/article/10.3390/s26154754/s1, File S1: Sample dataset.
